# The SRAQ-HP: Development and Initial Validation of a Tool to Assess Perceived Resource Adequacy Among Healthcare Professionals

**DOI:** 10.3390/ijerph22091380

**Published:** 2025-09-03

**Authors:** Olga Cerela-Boltunova, Inga Millere, Ingrida Trups-Kalne

**Affiliations:** 1 Department of Nursing and Midwifery, Riga Stradiņš University, LV-1067 Riga, Latvia; 2 Institute of Public Health, Psychology Laboratory, Riga Stradiņš University, LV-1067 Riga, Latvia; ingrida.trups-kalne@rsu.lv

**Keywords:** staffing adequacy, perceived workload, organisational support, quality of health care, personnel staffing and scheduling, health personnel, Latvia

## Abstract

Healthcare systems worldwide face growing challenges related to staff shortages, excessive workload, and deteriorating working conditions, which compromise both staff well-being and care quality. Despite these issues, there is a lack of validated tools that capture healthcare professionals’ subjective perceptions of resource adequacy. This study presents the development and initial validation of the Staff Resource Adequacy Questionnaire for Healthcare Professionals (SRAQ-HP), a multidimensional tool designed to assess staffing adequacy and workload, quality of care, and working conditions and support. The development process followed a mixed-methods design, incorporating theoretical foundations from Kanter’s empowerment theory, role enactment models, and professional competence frameworks. The initial item pool of 32 statements was reduced to 26 through expert reviews, focus groups, and pilot testing (*n* = 35). Content validity index (CVI = 0.931) and face validity index (FVI = 0.976) demonstrated high content relevance and clarity. Cronbach’s alpha for the full scale was 0.841, confirming internal consistency. Expert re-review confirmed strong content (S-CVI/Ave = 0.931) and face validity (FVI = 0.976) for the final 26-item version. Three core dimensions were retained: Staffing Adequacy and Workload, Quality of Care, and Working Conditions and Support. The SRAQ-HP provides a novel, evidence-based approach to systematically assess workforce sufficiency and support structures in clinical settings. It can guide decision-making in healthcare institutions and inform national workforce policies. Further research with larger and more diverse samples is needed to confirm its factorial validity and practical applicability.

## 1. Introduction

Today, healthcare systems around the world are facing significant challenges related to staff shortages [1], increasing workloads [2], the risk of professional burnout [3], and maintaining the quality of patient care [1]. Demographic changes [4], an ageing population [5], an increase in chronic diseases [6], and the impact of pandemics further exacerbate this situation [7]. In these circumstances, healthcare institutions must pay particular attention to how they plan, evaluate, and maintain their human resources. Effective staff planning, work environment assessment, and the introduction of professional support mechanisms are essential for ensuring patient safety and the quality of healthcare [8,9,10]. Patient outcomes are determined by both quantitative and qualitative factors, such as competence, emotional stability, decision-making ability, and the ability to work in an interdisciplinary team [11,12].

The common phenomenon of work overload, stress, and emotional exhaustion leads to high staff turnover, as illustrated by data from Latvia, where approximately 12% of nurses leave the sector each year [13,14]. In 2023, over 40% of nurses in Latvia regularly experienced overwork, while regional hospitals often had 30% fewer nurses on duty than planned [15]. Without adequate measurement, analysis and management of staff adequacy, the sustainability of the health system is seriously threatened [16].

In Latvia, as in many other countries worldwide, there is no systematically validated, localised health system tool available to assess healthcare workers’ subjective experiences in relation to staff adequacy, workload, and quality of care. Although various international scales [16,17,18,19,20] offer opportunities to assess professional competence, they rarely take into account the specifics of local healthcare systems, language, culture, and the psychosocial aspects of perceptions among staff. Unlike in Scandinavian countries, where both Nurse Professional Monitoring (NPM) [21] and revalidation tools exist, professional supervision in Latvia is limited to maintaining registration data.

Both in Latvia and abroad, the assessment tools used are often limited to keeping track of superficial indicators such as the number of patients per nurse or work schedule structure, ignoring the subjective experience of the staff [16,17,18,19,20]. Such tools do not provide a sufficient basis for making decisions about improving the working environment. The use of existing tools in Latvia is sporadic, with some institutions using fragmented assessments, but there is a lack of systematically validated tools [22]. During the COVID-19 pandemic, healthcare organisations lacked systematic and reliable tools for assessing the subjective experience of employees, making it difficult to respond promptly to shortages of resources and psycho-emotional stress [23]. This issue has become central to health policy and public trust in the wake of the pandemic.

A targeted and critical literature review was conducted to justify the development of the SRAQ-HP (Staff Resource Adequacy Questionnaire for Healthcare Professionals) tool. The reviewed tools include ASCOP (Actual Scope of Nursing Practice) [17], NWI (Nursing Work Index) [18], NPC (Nurse Professional Competence) [19], and the Misscare Scale [20]. While these tools provide information about the work environment, workload, and competence, they have the disadvantage of being unable to capture the local context, subjective perceptions, and organisational aspects simultaneously. The instruments reviewed in our conceptualisation phase address related but distinct constructs and therefore do not directly capture subjective staffing adequacy in our context. ASCOP focuses on the enactment and breadth of nurses’ roles and has clear strengths in delineating professional activities and task domains; however, it provides only an indirect view on resource sufficiency and does not quantify perceived adequacy of staffing in day-to-day practice [17]. NWI is widely used to assess the practice environment (e.g., leadership, participation in hospital affairs, foundations for quality care), offering robust evidence on organisational climate; yet it does not specifically target staff members’ subjective evaluation of staffing adequacy or workload balance within a unit [18]. NPC concentrates on competencies (knowledge, skills, attitudes) and thus informs professional preparedness, but it is not designed to evaluate perceived workload pressure or availability of human resources at the point of care [19]. Finally, the Misscare Scale sensitively captures missed or rationed care and is valuable as an outcome-oriented signal of strain; still, it does not assess the structural and organisational support conditions that precede such omissions nor staff’s felt adequacy of resources [20].

Rationale for SRAQ-HP. In summary, existing tools illuminate important adjacent domains (scope of practice, work environment, competence, and missed care) but do not jointly quantify staff’s subjective perception of resource adequacy together with workload and support in a way that is sensitive to local system features (language, culture, service organisation). The SRAQ-HP was therefore developed to fill this gap by integrating three theory-driven dimensions—Staffing Adequacy & Workload, Quality of Care, and Working Conditions & Support, and by providing locally adapted wording suitable for routine use in Latvian healthcare settings [17,18,19,20].

The SRAQ-HP was developed to address this issue. The development process involved an interdisciplinary team including healthcare practitioners, researchers, educational institution representatives, and data analysts. In the initial phase of the project, a much broader set of questions was developed, initially comprising more than 30 items covering various topics, such as the impact of organisational culture, communication between employees, emotional exhaustion, the number of patient complaints, shift frequency, work schedule flexibility, available resources, management support, and others.

Particular attention was paid to theoretical justification and empirical validity during the development. A three-dimensional model was chosen for analysing staff adequacy, the quality of care, working conditions, and support mechanisms. The theoretical basis is rooted in Kanter’s empowerment theory [24], Besner’s concept of role realisation in practice [25], and the professional competence models of the International Council of Nurses (ICN) and the World Health Organization (WHO) [26]. Each item of the tool was therefore formulated in relation to a specific theoretical approach to ensure content and construct validity.

The tool was developed in a context where indicators of employee well-being and organisational effectiveness were becoming increasingly important. The new tool will help to identify organisational weaknesses such as insufficient shift allocation of staff, lack of support, excessive workload, and emotional exhaustion. It will facilitate the setting of priorities for personnel planning, the monitoring of quality improvement initiatives, and the justification of investments in human resources.

In such circumstances, evidence-based tools that provide an accurate and systematic assessment of the current situation play a crucial role. Such data enables resource shortages to be identified, the performance of units and institutions at different levels to be compared, and essential support to be provided for decision-making, organisational change planning and strategies aimed at quality patient care and staff retention. Until now, human resource policy decisions in Latvian healthcare have often been based on limited and fragmented information, such as qualitative assessments, expert opinions, or one-off studies, rather than systematic, data-driven analysis [27]. This tool provides an opportunity to standardise the assessment of resource adequacy, an essential step towards evidence-based decision-making in workforce planning and improving care quality.

During the development process, particular emphasis was placed on taking an inclusive approach that considers the needs of managers, care workers, and researchers. The initial questions covered both objective indicators, such as the number of employees and the time taken to complete tasks, and subjective factors, such as emotional exhaustion, job satisfaction, trust in management, access to support mechanisms, and opportunities for professional development.

The study aimed to develop and conduct an initial validation of a multidimensional tool for assessing the subjective experience of healthcare professionals regarding the staff adequacy.

## 2. Theoretical Framework and Definition of Concepts

The theoretical framework is an essential element in the development of any new assessment tool. It provides conceptual clarity, helps define key research concepts, and establishes a logical connection between the issues addressed by the tool and its intended objectives [28]. Additionally, the theoretical framework serves as a guideline for developing items and provides a foundation for evaluating validity and reliability. As the issue of adequate staffing in healthcare involves objective and subjective aspects, the tool must be based on theories covering structural, psychological, organisational, and emotional dimensions [29].

Several theoretical models and concepts were integrated into the development of the SRAQ-HP to capture the reality of healthcare professionals’ work and ensure the multidimensional nature of the tool [24,25,26]. Structural Empowerment Theory, developed by Rosabeth Moss Kanter (1977), emphasises staff ability to access organisational resources, information, support, opportunities, and material resources [24]. This theory formed the basis of the Working Conditions and Support dimension, emphasising that staff well-being and effectiveness increase when they feel empowered and supported.

Besner and colleagues introduced the important concept of the difference between a formally defined professional role and the role actually performed in practice [25]. The organisational environment, management support, resource availability, and colleague cooperation determine factors that hinder or promote the full realisation of a role. This concept was used to define issues that assess staff ability to perform their duties to the fullest extent.

Both the ICN and the WHO offer comprehensive models for professional competence [26]. Here, competence is defined as a set of abilities comprising knowledge, skills, attitudes, and ethical values realised in professional practice. These models formed the basis of the SRAQ-HP tool across its three dimensions: Staffing Adequacy & Workload, Quality of Care, and Working Conditions & Support.

### 2.1. Selection of Models and Conceptual Justification

We anchored the SRAQ-HP in Structural Empowerment Theory (Kanter), the ICN professional competence frameworks, and WHO quality-of-care domains to ensure that the instrument captures how staff perceive whether resources are sufficient to enact their roles and deliver safe, person-centred care. Kanter’s model specifies four structural conditions, access to information, access to resources, access to support, and opportunities for professional growth—that jointly shape employees’ sense of being enabled to perform. While often studied in relation to job satisfaction, in clinical teams these four conditions are also proximal antecedents of perceived staffing adequacy: when access to material and social resources is constrained, staff experience the workload as misaligned with available capacity, i.e., “insufficient staffing”.

The ICN competence frameworks articulate the knowledge–skills–attitudes required to enact the professional role fully; thus, they inform the content domain in which adequate staffing and reasonable workload allow competent practice. The WHO quality-of-care framework (e.g., safety, effectiveness, timeliness, person-centredness) provides the reference for how resource adequacy translates into quality-of-care outcomes perceived by staff. Together, these models justify three theory-driven dimensions: (1) Staffing Adequacy & Workload, (2) Quality of Care, and (3) Working Conditions & Support. Operational scope of “Working Conditions & Support.” In this initial validation, Working Conditions & Support is operationalised as organisational and psychosocial support structures (e.g., managerial and peer support, scheduling practices, access to development and resources). Physical environmental factors (e.g., space, noise, ventilation, equipment layout) are recognised as important but were deliberately excluded from the scope of the present 26-item version and are planned for inclusion and validation in a subsequent, extended version of the instrument.

Four key concepts are clearly formulated in the tool, which are characterised by both theoretical definitions and empirical indications. The first of these is staff adequacy. Staff adequacy is an assessment, both subjective and objective, of whether a healthcare team has enough qualified staff at a given time and place to provide safe, high-quality, timely care [30]. The survey included questions about the number of nurses per patient, whether staff had to take over colleagues’ duties, and whether they were able to focus on each patient individually.

The second aspect is workload. This includes the quantity, intensity, and complexity of tasks within a given time frame, including both direct care activities and indirect activities such as documentation, communication with colleagues, and coordination [31]. Questions about subjective perceptions of workload, task distribution, and the impact of time constraints on the quality of care were included.

The third aspect is quality of care. Quality of care is defined as patient-centred, timely, safe, effective, and emotionally fulfilling care based on evidence-based practice [32]. This includes questions about staff perceptions of the adequacy of care, the feasibility of providing emotional care, and how staff shortages impact the quality of care.

The fourth aspect is managerial and organisational support. This includes feedback, involvement in decision-making, structured support mechanisms, and a psychologically safe environment [33]. It encompasses issues such as management’s response to work overload, opportunities for employees to express their views, and the availability of professional and emotional support.

Based on these concepts and theoretical frameworks, a structured, three-dimensional model was developed to serve as the basis for the items and their content logic. The model combines the concepts into thematic blocks, ensuring systematic coverage of staff perceptions of resources, working conditions, and quality of care. This three-dimensional structure is reflected in Table 1.

### 2.2. Theory-to-Dimension Mapping and Item Generation Procedure

We used a deductive, theory-driven content mapping to derive item pools for each dimension. Two authors independently coded initial statements to theoretical constructs with a priori coding rules:

- Items reflecting the balance between task demand and available human/material support, role-enactment feasibility, and task completion on time → Staffing Adequacy & Workload;
- Items reflecting safety, completeness, timeliness, emotional care, and the perceived impact of shortages on patient outcomes → Quality of Care;
- Items reflecting access to managerial/peer support, scheduling practices, opportunities for development, and a supportive climate → Working Conditions & Support.

Disagreements were resolved by consensus; items lacking clear theoretical alignment were reworded or removed prior to expert review and pilot testing. Table 1 summarises the three dimensions and their theoretical anchors; the final 26-item scale is presented later. Representative examples of the mapping are:

− “There are enough staff in our department during shifts …” (Item 1) → Staffing Adequacy & Workload;
− “The adequacy of staffing … improves the quality of care and patient safety” (Item 10) → Quality of Care;
− “Management responds actively to issues of work overload …” (Item 18) → Working Conditions & Support.

This transparent, theory-first mapping addresses content validity at the construct level and explains the rationale for selecting these three dimensions.

Each item in the questionnaire was constructed based directly on one of these theoretical conceptual sources. For instance, the statement ‘I have enough time to provide emotional support to patients’ represents the Quality of Care dimension and is rooted in Caring Theory. The statement ‘Management actively responds to work overload problems’ corresponds to Kanter’s structural support theory [24], while ‘There is sufficient staff in our department during the shift’ can be linked to the Role Enactment model, which emphasises objective assessment of adequacy.

In addition to the theoretical framework, an extensive literature review (N > 50 sources) was conducted to identify several recurring empirical relationships that strengthened the content base of the selected dimensions:

- Subjective workload often serves as a more accurate predictor of burnout than objective workload indicators [35];
- Management support is a critical factor in staff retention and maintaining professional quality [36];
- Emotional care aspects are generally rated lower in situations where available human resources are limited [37].

These theoretical and empirical findings highlight the need for a multidimensional tool that can capture organisational factors and the emotional reality of the staff as an integral part of healthcare quality.

Throughout this manuscript we consistently refer to the three theory-driven dimensions as Staffing Adequacy & Workload, Quality of Care, and Working Conditions & Support.

## 3. Methodology and Stages of Development

The SRAQ-HP was developed through a systematic, evidence-based process combining qualitative and quantitative data using a mixed-method approach. The aim was to create a standardised tool that is theoretically grounded and psychometrically robust, and which assesses the subjective experiences of healthcare staff regarding resource adequacy, workload, support mechanisms, and quality of care.

An interdisciplinary team comprising healthcare practitioners, university researchers, psychometrics specialists, and data analysts led the development of the tool. The process strictly adhered to ethical principles [38] and the internationally recognised stages of development of the tool [39,40], from conceptualisation to validation (seen Table 2).

### 3.1. Expert Panel: Selection Criteria, Diversity Targets, and Rating Anchors

Experts were invited based on (i) ≥5 years of clinical or managerial experience in healthcare; (ii) direct familiarity with workforce planning, quality, or education; and (iii) ability to appraise item relevance and clarity for front-line use. Exclusion criteria were <2 years of relevant experience or direct involvement in the item-writing team to avoid bias. Although our recruitment frame targeted multiple disciplines (nursing management, practising nurses, physicians, and public health/quality specialists), actual participation in this initial phase was concentrated in inpatient nursing management, as detailed below; multidisciplinary expansion is planned in the next round.

Diversity targets. To enhance generalisability, we set a priori targets to include experts across care settings (inpatient/outpatient/mental health), regions (Rīga/other), roles (manager/educator/front-line), and disciplines (nursing/medicine/public health). The present sample largely met role/setting targets within inpatient nursing but lacked physician and public health representation; this is acknowledged as a limitation and addressed in future sampling plans.

Rating anchors and decision rules. Before scoring, experts received a one-page calibration sheet with operational definitions (“relevant/clear”) and three annotated exemplars; a brief Q&A call harmonised interpretations, and residual disagreements were addressed during the post hoc item-revision step. For CVI (content validity), experts rated each item as 0 = not relevant or 1 = relevant (essential to the construct); we computed I-CVI and S-CVI/Ave. Decision rules were: I-CVI < 0.60 → remove; 0.60–0.78 → revise and re-evaluate; ≥0.78 → retain, subject to qualitative feedback. For FVI (face validity), experts rated 0 = unclear or 1 = clear (unambiguous, simple wording); items with FVI < 0.80 were revised. We also specify modified kappa (κ*) for I-CVI to account for chance agreement (thresholds: excellent ≥ 0.74; good 0.60–0.73; fair 0.40–0.59. These rules explain how low-performing items were removed or reworded and reconcile heterogeneous strictness among raters observed in Round 1.

Composition in this study. In Round 1, 10 experts (100% women)—predominantly inpatient nursing managers/educators from the Rīga region—completed CVI; in Round 2, 5 experts completed CVI + FVI. This composition reflects Latvia’s gender distribution in nursing and our initial recruitment focus; broader disciplinary participation is planned for subsequent validation rounds.

Initial piloting and qualitative work predominantly involved female practitioners from inpatient settings in the Rīga region. This composition reflects the current nursing workforce profile but limits generalisability to other disciplines and settings (e.g., physicians, outpatient and mental-health services, other regions). To address this, subsequent validation rounds will adopt a stratified, multicentre recruitment plan by region, care setting and role, explicitly including male practitioners and under-represented fields. Target sample sizes are N ≥ 200 for EFA and N ≥ 300 for CFA across multiple institutions.

### 3.2. Factor-Analytic Validation

To examine the latent structure of the SRAQ-HP, we conducted a two-stage validation in an independent, large sample of Latvian healthcare professionals: exploratory factor analysis followed by confirmatory factor analysis on a hold-out sample. Both analyses supported a correlated three-factor solution aligned with our theoretical dimensions (Staffing Adequacy & Workload; Quality of Care; Working Conditions & Support). Full methods, model comparisons, and diagnostics are reported in a companion paper.

Ethical principles were observed throughout the process of developing and testing the tool [38]. The study design was approved by the RSU Ethics Committee, all participants gave informed consent; however, the data were analysed anonymously and processed in accordance with the General Data Protection Regulation (GDPR) requirements [41].

### 3.3. Statistical Reporting Conventions

Given the ordinal origin of single Likert items but the near-interval behaviour of multi-item subscale scores, we report subscales as Mean (SD), with 95% confidence intervals, and provide Median (IQR) as a sensitivity descriptor. Normality (Shapiro–Wilk) and homoscedasticity were checked; where assumptions were questionable, we additionally considered robust summaries (Median, IQR) and, for group contrasts, Welch tests or non-parametric alternatives. Internal consistency is reported as Cronbach’s α (dimension-wise) and, where feasible, McDonald’s ω with 95% CIs (bootstrap). For content/face validity we present CVI/FVI as proportions (0/1 anchors) and reference modified kappa (κ*) to account for chance agreement. All tests are two-tailed.

In conclusion, it should be emphasised that the initial version of the SRAQ-HP tool shows promising psychometric properties that meet accepted psychometric criteria, including adequate internal consistency and theoretical structure. The tool was developed in accordance with the methodological principles, involving experts and representatives of the target group. These results indicate good initial content and construct validity. However, further research involving larger and more diverse samples is required to draw comprehensive conclusions regarding the practical applicability of the tool. Future research should include detailed factorial validation (e.g., exploratory and confirmatory factor analysis), criterion validity testing (by comparison with objective workplace indicators such as patient satisfaction or staff turnover) and construct validity testing (by comparison with similar tools such as the CBI or PES-NWI). Only after these additional analyses have been carried out will it be possible to fully assess the reliability, generalisability, and practical applicability of the SRAQ-HP tool in assessing human resources in healthcare institutions. The current results provide a basis for further development and reinforce the tool’s potential as part of an evidence-based approach to human resource planning in healthcare.

## 4. Interpretation of the Scale

Likert-type scales are usually questionnaires comprising several items and a set of response options ranging, e.g., from ‘never’ to ‘always’. These are often summed or averaged to obtain a total score. To make the results interpretable in practice, researchers often divide this total score into qualitative categories such as ‘very low’, ‘low’, ‘medium’, and ‘high’ [42]. There are several methodological approaches to determining the thresholds between these levels:

- Expert evaluation [43];
- Normative data (comparative standards) [44];
- Empirical distribution in a pilot study [45];
- Criterion-based thresholds [46].

The interpretation levels for the SRAQ-HP scale are based on a multi-level methodological approach combining theoretical, empirical, and practical aspects. As there are no universally standardised thresholds for Likert-type scales, several scientifically based strategies were employed to develop reliable and practical assessment levels [42,43,44,45,46]. Initially, the structure of the interpretation scales was developed based on theoretical foundations and approaches used in the international literature on tools such as the CBI [47], MBI [48], and PES-NWI [49], in which the level classification was developed with the help of experts or through the analysis of empirical data distribution. This approach ensures compliance with proven and validated psychometric practice. In parallel, expert consultations were organised involving healthcare professionals with experience in personnel management and research. These experts were tasked with helping to define thresholds that realistically reflect significant changes in staff perceptions of resource adequacy and quality of care. The experts identified the average ratings that would be considered optimal, acceptable, or critical, taking into account staff workload management and management support availability. This was followed by an empirical phase, during which the proposed levels were compared with the actual data distribution obtained from both the pilot and main studies. The histograms, dispersion, and central tendency analysis of the results provided an insight into how proportionally the respondents were distributed across the different points on the scale, as well as the value ranges in which significant accumulations were formed. To ensure simplicity and consistency of interpretation, a simple mathematical calculation was used to assign each of the five rating categories an interval of approximately 0.80 on a scale from 1 to 5. This method is widely used in practical research, particularly when normative data or results from ROC analysis are not yet available [46]. The interpretation levels were adjusted according to the data distribution and expert recommendations, ensuring they were mathematically symmetrical and semantically meaningful.

The current cut-off values are considered an initial standard, providing guidance for self-assessment by institutions and offering practical utility. It should be emphasised, however, that these levels do not yet have any clinical or normative significance and require further validation. Planned future studies will use ROC analysis to determine optimal thresholds in relation to external criteria, such as burnout and patient satisfaction. Quartile and z-score analyses will also be used to construct a normative distribution. Additionally, data must be collected again across different institutions and contexts to strengthen the comparability of the scale and standardise cut-off values for future use. In summary, this combined approach—starting with a literature- and expert-driven structure, complemented by empirical adaptation and validation planning—provides a reliable basis for developing the levels of interpretation. This enables the SRAQ-HP tool to be used as both a data collection tool and an evidence-based mechanism for assessing and improving the healthcare work environment.

The questionnaire is based on a 5-point Likert scale: Completely disagree (1); Disagree (2); Neutral (3); Agree (4); Completely agree (5). This provides easily interpretable data on staff satisfaction and resource adequacy.

Scores are averaged per subscale; higher values indicate better staffing adequacy, working conditions and quality of care, while lower values flag potential risk. The ‘Staffing Adequacy and Workload’ section assesses whether staff consider human resources to be sufficient to ensure a balanced workload. Table 3 shows the scale rating levels.

The ‘Quality of Care’ section reveals how employees assess the impact of adequate staffing on quality of care and patient safety. Table 4 shows the scale rating levels.

The ‘Working Conditions and Support’ section assesses how effectively management provides support to the staff, and the impact of this on their ability to perform their duties adequately. Table 5 shows the scale rating levels.

After obtaining the average results in each section, these can be used to draw general conclusions about the adequacy of human resources and their impact on the quality of care in the hospital (see Table 6).

Average scores can also be analysed by department or job title to identify groups with the greatest need for additional resources or support. Repeating the survey regularly makes it possible to observe changes in employee attitudes and identify trends to help management make long-term decisions about resources and work organisation.

The results can be compared with other data, such as employee turnover, sick leave, and patient satisfaction indicators, to understand the relationship between adequate resources and the organisation’s overall effectiveness. The data is analysed to identify trends and potential problems in each section. This helps management determine whether improvements are needed in staffing or working conditions.

This questionnaire has been developed to assess how effectively hospitals and healthcare institutions provide the necessary human resources and working conditions to ensure quality patient care. The tool helps identify the main challenges faced by healthcare staff and analyse the adequacy of resources and support affecting the quality of care and staff well-being. It focuses specifically on assessing the adequacy of resources in relation to quality of care, workload, and management support. This allows for a very specific focus on the availability of resources and staff support rather than general working conditions or satisfaction. This specific approach may be more effective than some international tools, which cover a broader range of topics but can sometimes lose focus on specific aspects of resources and quality of care.

The questionnaire is designed to assess three main aspects. Staffing Adequacy and Workload gauges staff perceptions of the workload, staffing levels, and whether they have the necessary resources to perform care tasks effectively. 9 statements. The Quality of Care section analyses the impact of human resource adequacy on the quality of care and patient safety. 7 statements. The Working Conditions and Support section assesses the support provided by management and the working environment, which affects staff resilience and satisfaction. 10 statements.

Potential usage scenarios have been developed. The questionnaire can help hospital management to identify departments or shifts where staffing levels need to be increased or workloads optimised. The questionnaire provides data on employee satisfaction with the work environment and management support, which can serve as a basis for planning improvements. The questionnaire allows to assess the impact of resource adequacy on the quality of care and patient safety, enabling management to take corrective action.

The scale has certain advantages and limitations. The positive and negative aspects are presented in Table 7.

## 5. Results

The Results section provides a detailed overview of the development, adaptation, and psychometric validation of the SRAQ-HP tool. The obtained data reflect the results of each development stage, including CVI and FVI assessments, pilot study results, and psychometric analysis indicators from the main study. Both qualitative and quantitative methods were employed in the analysis to ensure content validity, reliability, and construct validity. The results are organised chronologically according to the stages of the development process.

Four representative groups of respondents were analysed within the study. In total, there were 10 experts who performed the initial CVI of the scale, 10 focus group participants who took part in a discussion on the content and understanding of the tool, 35 respondents in a pilot study who completed the initial version of the scale, and 5 experts who, in addition to CVI, also assessed the clarity and discrimination indicators (FVI) of the tool.

The first ten experts were all women (100%), with an average age of 45.2 years (min = 34 years; max = 59 years) and an average of 17.4 years’ experience working in healthcare. Most of the experts represented medical institutions in the Riga region (80%), while the rest came from other regions of Latvia. All of the experts had a higher education qualification in healthcare and 90% held managerial positions such as head nurses, managers, or teaching staff. 60% of the experts reported a total workload exceeding 1.0 FTE, while 30% reported shift work. All of the experts worked in inpatient care settings and 90% were also involved in other duties such as methodological work, staff training, or quality control.

The focus group participants were also all women, with an average age of 38.6 years and an average of 12.1 years work experience. In Latvia, most nurses are women, so it is common for women to be in the majority. 100% of the participants were from Riga and 90% of the focus group participants had higher education, but the distribution of positions was more diverse: 50% held managerial positions, while the remainder were senior nurses or experienced practising nurses. Relatively fewer respondents worked with a total workload above 1.0 FTE (40%) or outside normal working hours (60% worked shifts). The work environment was inpatient care in 90% of cases and 70% of respondents were involved in additional professional development activities.

The main group of respondents (*n* = 35) who participated in the initial scale completion were women (100%). The group’s average age was 35.6 years (min = 22; max = 65), and the average length of service was 9.46 years (min = 2; max = 40). 65% of the respondents were from the Riga region, while the rest were from other regions. 85% of the respondents had higher education, but only 20% held managerial positions. Only 30% of the respondents had a workload exceeding 1.0 FTE. However, 80% worked shifts and 88% indicated that they worked in inpatient care. Additional responsibilities were reported by 60% of the respondents.

The additional experts (*n* = 5) involved in both the CVI and FVI assessments were also 100% women. They had an average age of 41.0 years and an average length of service of 14.8 years. They all represented inpatient facilities in Riga, had a higher education, and held managerial positions. Of this group, 80% worked with more than 1.0 FTE workload, but only 20% indicated that they worked shifts. 100% of the respondents were involved in professional development or training activities.

To assess the compliance of the developed SRAQ-HP items with the proposed theoretical dimensions, a CVI calculation was performed based on an assessment by 10 experts. This process was carried out in accordance with internationally recognised policy and tool development recommendations [50]. In Round 1 (CVI), I-CVI ranged 0.531–0.719; following decision rules, 3 items were removed and 9 reworded. In Round 2, CVI/FVI indicated improved relevance and clarity (S-CVI/Ave = 0.931; FVI = 0.976). The results are presented in Table 8.

The overall CVI for the entire scale was 0.653. The CVI of the experts ranged from 0.531 to 0.719. The lowest ratings were given by Experts 1 and 6 (CVI = 0.531), which are below average. Perhaps they were stricter in their assessment or misunderstood the criteria. Experts 2, 4, and 9 gave moderately high ratings ranging from 0.62 to 0.72. Experts 3, 5, 7, 8, and 10 gave high ratings that were consistently positive with a CVI of 0.719.

When the CVI was reviewed for each statement in the survey, it was found that items 1, 8, 11, 14, and 21 received the highest ratings, with full consistency among the experts (CVI = 1.0). Of course, there were some problematic items. Items 3 (CVI = 0.3), 19 (CVI = 0), and 30–32 (CVI = 0) raise significant concerns as they are below the validity threshold. Items 12 and 18 (CVI = 0.4) are also considered problematic and may need to be excluded or clarified. Items with a CVI of 0.5 and 0.6 were also reviewed to ensure the reliability and accuracy of the questions.

Taking the obtained data into account, the problematic questions (3, 5, 12, 18, 19, and 29) were reviewed by analysing their wording and rephrasing them to better suit the respondents. Based on the analysis, questions 30, 31, and 32 were removed from the scale. No new questions were added, since all existing questions reflect the essence of the survey qualitatively. Questions that had a positive impact (e.g., questions 6, 13, 14, and 20) were left unchanged because they improve the overall quality of the scale.

To supplement the quantitative content validation process and ensure the semantic and conceptual relevance of the initial SRAQ-HP scale to the clinical context, a focus group discussion was organised during the tool development phase. The focus group served as a qualitative validation method, allowing for a deeper understanding of healthcare professionals’ perceptions of the concept of staff adequacy and assessing the relevance of the proposed items to real-life practice. The discussion aimed to identify unclear or overlapping wording, clarify the appropriateness of the language, and ensure each clause reflected relevant practical aspects.

Ten healthcare professionals were selected for the focus group using a targeted sampling approach. The participants included five nurses, two head nurses, two healthcare managers, and one university lecturer with experience in nursing education and healthcare research. Such diversity ensured that views from different levels of the healthcare system were obtained.

The discussion took place on Zoom in November 2024 and lasted approximately 90 min. It was organised according to a semi-structured interview scenario that included open-ended questions about staff workload, management support, emotional care options, and interprofessional collaboration. Written informed consent was obtained from all participants, and the discussion was recorded, transcribed, and anonymised.

The results showed a high level of overall agreement and demand for this type of tool. The participants stated that there are currently no structured tools available that enable managers to objectively assess subjective perceptions of staff adequacy. Several improvements to the wording of the items were suggested, including the simplification of technical language, the revision of negatively worded statements, and the use of more specific terms. For instance, the statement ‘During shifts, our department has sufficient staff’ was linked to the need for a clearer distinction between objective and subjective perceptions of adequacy. Participants also recommended adding a dimension to the tool that would cover interprofessional collaboration, as several of them emphasised that ‘the availability of doctors and the team’s response affect our ability to provide effective care’.

The focus group results were systematised and used as a basis for revising the items prior to the quantitative content validation phase. This ensured that the items included in subsequent development stages were conceptually appropriate, relevant in practice, and linguistically accurate. The contribution of the focus groups to the development of the tool significantly improved the validity and usability of the SRAQ-HP in clinical practice.

Following content validation and initial focus group discussions, the next stage of the tool’s development involved an empirical evaluation of the items to analyse reaction indices (item difficulties) and discrimination indices. This stage involved 35 healthcare professionals from different regions and medical specialties who filled in the initial version of the tool. Respondents’ ages ranged from 22 to 65 years, with an average age of 35.6 years (SD = 11.74). The length of service in healthcare ranged from 2 to 40 years, with an average of 9.46 years (SD = 8.98).

The reaction index (mean item endorsement) was calculated as the average rating of each question, while the discrimination index (item-total correlation) was calculated as the correlation between each item’s rating and the total scale score (excluding that item). These indicators helped to determine how effectively each item distinguished respondents with high and low perceptions of staff adequacy. The reaction and discrimination indices are shown in Table 9.

Overall, the questionnaire consists of three dimensions. The internal consistency analysis revealed that Cronbach’s alpha was 0.620 for the first dimension, 0.817 for the second, and 0.888 for the third. The overall Cronbach’s alpha value for the entire scale is 0.841, indicating good reliability. While the alpha value for the first dimension is relatively low, it is still considered acceptable for initial studies. This may indicate a need to revise individual items or refine the content focus of the dimensions. Further analysis with a larger sample is recommended in subsequent stages of development to strengthen the psychometric properties of this dimension and possibly improve its internal consistency.

The reaction index (mean) measures the average response of respondents to each question. It provides information on how high or low the average reaction to the content of the question is. The Likert 5-point scale reaction index reflects the average rating given by respondents to a question, where 1 is the lowest rating (‘completely disagree’) and 5 is the highest rating (‘completely agree’). Results ranging from 1.8 to 4.2 cover a wide range, allowing for detailed interpretation of the respondents’ answers.

A low reaction value (mean = 1.8–2.4) indicates low agreement; in some cases it may reflect reverse-wording, limited relevance, or a mismatch with respondents’ context. For example, the question may not correspond to the respondent’s situation, or the question may be negatively worded, or the respondents may consider the topic unimportant. When analysing the *n* = 35 group, questions 1, 3, 5, 21, and 27 corresponded to the given limits.

A moderately low reaction value (mean = 2.5–3.0) indicates neutral or slightly negative responses. Respondents may be undecided or consider the question partially irrelevant. Questions 2, 4, 8, 16, 20, 22, 24, 26, 28, and 29 corresponded to these limits for the *n* = 35 group.

Moderately high reaction value (mean = 3.1–3.8). This shows a positive assessment or tendency to agree. The question is fairly well accepted and correlates with the overall conception. Questions 7, 9, 10, 11, 12, 19, 23, and 25 corresponded to these limits for the *n* = 35 group.

A high reaction value (mean = 3.9–4.2) indicates strong agreement or high evaluation. These questions are usually clear and appropriate. Questions 6, 13, 14, 15, 17, and 18 corresponded to these limits for the *n* = 35 group.

Corrected Item-Total Correlation, also known as the discrimination index, measures how well each question correlates with the total scale when the question itself is excluded. This metric allows problematic questions to be identified. The discrimination coefficient ranges from −1 to +1. A positive coefficient indicates that respondents with higher values on the scale answer this question affirmatively. A negative coefficient indicates that respondents with lower values on the scale answer this question affirmatively. When analysing the *n* = 35 group, all the indicators were positive, ranging from 0.104 to 0.631.

The optimal discrimination index ranges from 0.2 to 0.8. Values below 0.2 indicate a low correlation, meaning the question does not correspond well to the overall scale concept. Values between 0.2 and 0.5 reflect an average correlation—the question is acceptable but could be improved. Values above 0.5 indicate a good correlation and show that the question corresponds well to the overall scale. The analysis of the data revealed the following problem questions: Question 3 (0.181), Question 5 (0.139), Question 12 (0.062), Question 16 (0.116), Question 18 (0.104), and Question 29 (0.108). These questions correlated poorly with the overall scale, and the low correlation may indicate an issue with the wording or context. Seven questions were highly discriminative (with a score above 0.5): Question 11 (0.580), Question 13 (0.631), Question 14 (0.510), Question 21 (0.544), Question 22 (0.558), Question 23 (0.523), and Question 28 (0.545).

A combined analysis of the lowest reaction and discrimination indices revealed which questions were the most problematic, as they do not correspond to respondents’ experiences and were inconsistent with the overall scale. Question 3 had a very low reaction (2.09) and discrimination (0.181). This question is inappropriate and should be removed or reworded. Question 5 was weak in terms of both reaction (2.20) and discrimination (0.139) and should be reworded or removed. Although the reaction to Question 12 was average (3.26), the discrimination was very low (0.062), thus the question should be re-evaluated. Question 18 had a high reaction (4.09), but very low discrimination (0.104), indicating a problem with the wording. Question 29 had a low reaction (2.23) and low discrimination (0.108), which significantly weakens the overall reliability.

Standard deviation (SD) shows the variation in responses. Higher SD indicates greater variation, meaning that respondents answered differently, while lower SD indicates unanimity. Question 4 (SD = 1.323), Question 9 (SD = 1.431), and Question 28 (SD = 1.350) all showed high SD (above 1.3). Questions with high variation may indicate various opinions or different understandings. Question 6 (SD = 1.051) and Question 13 (SD = 1.014) showed low SD (below 1.1). Questions with low variation may be too unambiguous, reducing scale discrimination. The sum of the results (Sum) shows the total number of points for each question from all respondents. This indicator reflects the extent to which a particular question has been assessed overall and can be used to identify questions with extremely low or high ratings. Question 3 (73), Question 5 (77), Question 21 (78), Question 27 (78), and Question 29 (78) showed the lowest values. These questions could weaken the overall scale and should be re-examined with particular care. Question 6 (144), Question 13 (141), Question 14 (145), Question 17 (141), and Question 18 (143), on the other hand, were popular among respondents, being clear and meaningful. However, it should be analysed whether these questions provide sufficient discrimination (e.g., Question 18).

Cronbach’s Alpha if Item Deleted’ shows how the overall Cronbach’s Alpha would change if a specific question were removed. An increase in the Alpha value after removing a question indicates that the question has a negative impact on overall reliability. This indicates that the question may need to be removed or reworded. If the Alpha value remains unchanged or decreases, this indicates that the question positively contributes to the overall reliability and should be retained. Questions 6, 9, 13, 14, and 20 would reduce the Cronbach’s Alpha value if they were removed. This indicates that these questions strengthen the overall reliability and should be retained in the scale. Question 7 (0.840), Question 17 (0.837), and Question 24 (0.835) had a neutral effect on Cronbach’s alpha. These questions would have a minimal effect on the Alpha value if they were removed. They may be acceptable on the scale but could be improved. Question 3 (0.841), Question 5 (0.843), Question 12 (0.846), Question 18 (0.844), and Question 29 (0.844) would increase the Cronbach’s Alpha value if they were removed. This indicates that these questions reduce the overall reliability and should be re-evaluated or removed from the scale.

Squared Multiple Correlation (SMC) indicates the extent to which a particular question can be explained by the other questions on the scale. SMC measures how well the question fits the overall concept and its ability to discriminate between respondents. All questions had an SMC above 0.777. Question 15 had the highest SMC (0.958).

Taking into account all data in Table 5, the problematic questions were: Questions 3, 5, 12, 18, and 29. Questions that worked well and had a positive impact on reliability: Questions 6, 9, 13, 14, and 20. Questions with good discrimination (above 0.5): Questions 11, 13, 14, 21, 22, 23, and 28. Questions that worked moderately well and have potential for improvement: Questions 7, 17, and 24.

After determining the CVI, reaction, and discrimination indices, the following questions were removed: Questions 3, 5, and 18. The following questions were revised: Questions 12 and 29 We improved the wording of the questions with average performance. Questions 7, 17, and 24. We reviewed questions with a low standard deviation (SD) to achieve greater variation: Questions 6 and 13.

Taking this psychometric evaluation into account, a new version of the scale was created which only includes items with adequate statistical indicators and theoretical significance. This improved version of the scale is shown in Table 10 and was used in subsequent stages.

After the revision of the initial survey scale a second version containing 26 points was made. This scale was presented to five experts who met the inclusion criteria for completing the survey, in order to assess the FVI and CVI. Table 11 below shows the results of evaluating each item of the scale, the total number of points, and the CVI indicators.

The overall average CVI for all items is 0.931, which indicates high content validity. The final version, which has a CVI of 0.931, proves that the scale redesign was successful. The initial review showed heterogeneous item-level CVI (I-CVI range 0.531–0.719); after revision, the S-CVI/Ave = 0.931. The final version (CVI = 0.931) had high content validity, indicating that the questions were clearer and more relevant.

Content validity was ensured using the content validity index from expert evaluations. The individual CVI (I-CVI) for all statements included exceeded the traditionally recommended threshold of 0.78, meaning that the experts considered them to be appropriate for assessing the target construct [51]. The average CVI (S-CVI/Ave) calculated for the scale as a whole was also high (almost 0.9), confirming that the expert panel generally rated the scale items as highly relevant [51]. The CVI is critical in scale development as it indicates whether the included items cover the essential aspects that the scale is trying to measure [51]. In this study, the high CVI scores indicate that the SRAQ-HP scale covers the main aspects of human resource adequacy in healthcare and that no significant content gaps were identified in any of the retained questions. Some initially proposed questions with lower expert ratings (e.g., questions that duplicated the content of other items or were ambiguous) were excluded prior to the pilot study. This ensured that only valid statements were included in the pilot study.

Five experts participated in the FVI determination process. When measuring the FVI, the experts were asked the following question: ‘Did you understand what each point meant when you completed the Staff Adequacy Scale?’ Each expert rated the clarity of each item (0 = unclear, 1 = clear). The results are presented in Table 12.

The overall FVI was 0.976, indicating that the majority of assessed criteria are appropriate for evaluating resources. This suggests a high level of understanding of the tool and its suitability for the intended users. Only a few items (12, 14, and 24) received a rating below the maximum value (FVI = 0.8), suggesting possible ambiguity or differences in understanding these items. These items may need to be clarified or reworded. While the ratings were similar, Experts 2–4 had FVI = 0.961, whereas Experts 1 and 5 = 1.000.

Suitability of the tool for use, given that the overall FVI was very high, it can be concluded that the tool’s assessment of staffing adequacy and the clarity of item wording were well received by the expert panel.

### Brief Factor-Analytic Findings

Data were suitable for factor analysis (KMO = 0.892; Bartlett’s χ^2^ (325) = 33,046.524, *p* < 0.001). In an independent large-sample CFA (*n* = 1369), a correlated three-factor model showed high comparative fit (CFI = 0.968; TLI = 0.964); full model diagnostics (including RMSEA/SRMR and robustness checks) are reported in the companion paper.

## 6. Implementation and Operating Procedures

The SRAQ-HP is intended for quarterly administration at unit level (optional biannual cadence in stable units). Anonymous, online or paper administration to all staff on duty within a two-week window, minimum 60%-unit response rate is recommended for reliable unit-level estimates.

Dimension scores are the mean of item responses (1–5). We report Mean (SD) with 95% CIs; as a sensitivity check, Median (IQR) may be added.

Results are compiled into a unit dashboard with three subscale means and 95% CIs, compared with historical values and hospital benchmarks. Feedback to unit leaders within 30 days of close and leaders can discuss results with staff.

Units with any subscale <3.0 or a ≥ 0.3 decline vs. prior cycle trigger a brief action plan (e.g., staffing adjustments, schedule redesign, peer-support/supervision), followed by a repeat measurement next quarter. Only aggregated results are shared beyond the unit level; no individual-level reporting.

Following a quarterly SRAQ-HP, Unit A showed Working Conditions & Support = 2.8 (95% CI 2.6–3.0). Management introduced fortnightly peer debriefs and adjusted shift overlaps. At the next cycle, the subscale rose to 3.4, while Staffing Adequacy & Workload remained stable.

## 7. Discussion

The Latvian healthcare system has long suffered from human resource problems, particularly a shortage of qualified nurses and doctors. Statistics show that, for example, there are only ~4.2 nurses for every 1000 inhabitants in Latvia, which is less than half of the EU average (~8.5 per 1000) [52]. This situation limits the availability of services and even threatens the successful implementation of health reforms [52]. In practice, this means that, in many hospitals, existing staff are forced to work excessive hours to fill vacancies. Nurses often ‘fill in’ for absent colleagues, resulting in sleepless nights and burnout syndrome [53,54]. In the long term, such work overload can lead to professional burnout and a decline in work quality, causing some staff to leave the sector [54,55]. As patients may not directly experience the effects of staff shortages in the short term, there is a risk that this issue will be overlooked or underestimated at a systemic level [52,56].

Until now, staff planning decisions in Latvia have mainly been based on quantitative indicators such as the number of staff positions, budget costs, and short-term solutions rather than on the assessments of staff subjective experiences and working conditions [52,57]. Healthcare reforms have often focused on optimising spending rather than staff well-being. For instance, during periods of budgetary constraints, the focus has typically been on reducing costs rather than improving staff well-being and the working environment [58]. This approach is dangerous because ignoring staff needs undermines the system’s long-term capacity [13,54]. International studies show that in health systems where management focuses excessively on performance indicators and cost savings rather than staff support, the risks of burnout and staff turnover increase [58]. Until now no unified tool has been developed in Latvia for regularly and systematically measuring healthcare professionals’ satisfaction with human resource adequacy and their subjective workload and work experience. Consequently, management lacks evidence-based data on staff sentiment and the adequacy of resources on the front line. Decisions on personnel policy are often made without sufficient information or based on isolated incidents and complaints rather than systematic data analysis.

Given this, the introduction of the SRAQ-HP tool fills a significant gap in Latvia’s healthcare data ecosystem. The SRAQ-HP has been developed to assess staff subjective views on aspects of the work environment that could not previously be quantified. This tool provides the first opportunity to measure subjective workload, opportunities for professional role fulfilment, and the level of management support in everyday work objectively and in a standardised way. Initial research results confirm the high potential of the tool. The SRAQ-HP survey has been found to have sufficient internal consistency and content validity, indicating that the questions in the questionnaire are reliable and fit for purpose. The consistency and reliability of the tool, such as high Cronbach’s α values, indicate that the survey reliably measures the intended dimensions and can serve as a reliable source of data for further analysis. While it is not necessary to list all the quantitative indicators again here, it should be emphasised that validating the questionnaire in the Latvian context confirms its usefulness. Respondents generally indicated that they feel the significant impact of workload and resource shortages in their daily work, which aligns with long-standing concerns in the sector. In other words, SRAQ-HP data empirically confirm issues that were previously known mainly through anecdotal experience. This tool brings the hidden to light, quantifies subjective fatigue and overwork, and identifies where management support is most lacking. The SRAQ-HP therefore provides an important basis for a more informed discussion on human resource policy in healthcare.

The importance of the tool is also confirmed by the context in which it was developed: the COVID-19 pandemic and the post-pandemic period have exacerbated workload and burnout issues for staff worldwide [59,60]. In Latvia, the pandemic revealed how critical it is to have sufficient and motivated staff [27,61]. Unfortunately, the absence of objective data on staff workload and satisfaction meant that only fragmented solutions were adopted after the crisis, such as short-term bonuses and campaign-style recruitment. These do not solve systemic problems [57]. SRAQ-HP can serve as an early warning system by regularly collecting data to identify critical increases in workload or staff dissatisfaction, which would otherwise only become apparent at a late stage—for example, through mass resignations or strikes. In summary, introducing this tool is a step towards developing an evidence-based personnel policy. This could mark a turning point for the Latvian healthcare system, shifting the focus from crisis management to proactive monitoring and planning of the working environment.

The situation in Latvia with regard to staff resource adequacy and the use of data in decision-making should be compared with international best practice, particularly in countries with well-developed healthcare systems where workforce planning is data-driven. The Scandinavian countries are a good benchmark in this regard. Finland, for example, has been using the RAFAELA system, a special tool for hospital staff planning and workload management, since the 1990s [62]. RAFAELA involves the daily measurement of patient care intensity and links this to the number of nurses required to provide an adequate level of care. The system is based on three elements: a patient classification tool, information on available human resources, and a professional assessment of the optimal workload (the PAONCIL method) [62]. This allows for the daily monitoring of whether there are enough staff to provide quality care for a given patient volume, providing immediate feedback to managers. Consequently, this system is now used in almost all Finnish hospitals, with similar approaches being introduced elsewhere in Europe and Asia [62]. The widespread use of the RAFAELA system in the Nordic countries confirms that data-based workload management is being taken seriously, with the system becoming the standard for determining optimal staffing levels rather than relying solely on intuitive management decisions [63]. RAFAELA also serves as a professional monitoring system that enables benchmarking of departmental work intensity between hospitals and over time, facilitating data analysis and targeted improvements. Other tools based on a similar philosophy include the Safer Nursing Care Tool used in British hospitals, which helps determine the number of nurses needed per shift based on patient care intensity data [64]. This tool is also effectively a system for monitoring appropriate workloads and staffing levels. It is widely used in NHS hospitals to ensure patient safety.

In OECD countries, public sector employers are increasingly organising regular surveys to measure staff satisfaction and working conditions [65]. Research shows that governments in many countries are introducing staff surveys to improve performance and service quality. The most popular focus of these surveys is staff job satisfaction [66]. In several countries, survey questions focus on staff’s subjective views of the working environment, as there is recognition that staff engagement and well-being directly impact organisational performance [65]. For example, the US Office of Personnel Management conducts an annual survey of federal employees to monitor perceptions of working conditions in the civil service [67]. This is a familiar practice for many healthcare institutions, with hospitals and clinics regularly surveying their staff about their perception of workload, support from management, and satisfaction. They then use this data to plan interventions to improve staff well-being [68,69,70,71]. Staff feedback thus becomes a valuable resource for organisational management, enabling targeted improvements to be initiated based on the real needs and recommendations of staff [65]. Latvia has lagged behind in this regard, as there have been neither regular nationwide hospital staff surveys nor an integrated system for collecting workload data. Therefore, introducing SRAQ-HP will bring Latvia closer to the good practices of OECD countries and ensure that the voice of employees is heard at the policy level.

It is important to note that in other countries there are also official staff monitoring systems in place at the national level. For instance, Finnish Institute for Health and Welfare (THL) collects and analyses data on the healthcare workforce in both the public sector and overall. This includes monitoring the adequacy of the number of nurses and doctors, as well as tracking their migration [72]. Other Nordic countries also carry out regular health workforce forecasting and monitoring activities. Norway, for example, conducts Arbeidsgivermonitoren surveys to analyse the situation of public sector employees, including those in healthcare [73]. Frameworks have been developed at OECD [64] and WHO [60] levels to encourage countries to develop human resource monitoring mechanisms. For example, the World Health Organization’s Global Strategy on Human Resources for Health: Workforce 2030 emphasises the need to strengthen health workforce data by introducing National Health Workforce Accounts and other tools to ensure regular data collection and analysis for human resource planning [74]. All of this demonstrates that the international approach is shifting towards data-driven methods, both at the micro level in hospitals, where tools such as RAFAELA [62] and regular surveys are used, and at the macro level, where national and international monitoring systems are employed.

Comparing the situation in Latvia, we have one of the lowest ratios of nurses and doctors per capita in Europe, and at the same time, there has been a lack of modern tools to manage this problem [54,56]. In Scandinavia, where healthcare is better, active investments are being made in monitoring and improving working conditions. This recognises that even adequate staffing is meaningless if employees are overworked or dissatisfied [62,72,73]. Following these examples, the Latvian healthcare system should not only use SRAQ-HP as a research tool, but also integrate it into daily management processes, as the Nordic countries have done with their monitoring tools. This will gradually bring us closer to the international standard, where decisions are based on data and the staff perspective is systematically taken into account.

One innovative aspect of the SRAQ-HP tool is its inclusion of multiple dimensions in a complex assessment. The survey covers subjective perceptions of workload, aspects of professional role fulfilment, and management support. When discussing the integration of these dimensions, it is important to analyse why each one is important and how they together form a holistic picture of the state of the workforce.

Objective indicators, such as the number of patients per nurse, do not always reflect how employees perceive their workload themselves [75]. Two identical departments with a similar number of patients may experience different subjective workloads depending on factors such as the severity of the patients, the organisation of the team’s work, technical support, and so on [76]. Studies confirm a strong link between an excessive workload and burnout as well as decline in the quality of care [69]. For instance, structured studies have revealed [77,78,79] that high work intensity promotes the implied rationalisation of care, whereby nurses unintentionally omit less critical activities due to time constraints, thereby worsening the overall quality of care. Another consequence is increased emotional exhaustion. Employees who constantly work beyond their capacity lose motivation and become emotionally numb, which negatively impacts their health and the patient experience [79,80,81]. Staff fatigue and dissatisfaction caused by high workloads have been found to reduce patient safety and the quality of care. For example, systematic reviews have found a correlation between nurse burnout and lower patient satisfaction and safety indicators [82]. Therefore, the Subjective Workload dimension within the SRAQ-HP provides a vital signal: if survey data consistently show that staff in a particular department report very high workloads, this indicates the need for immediate action to prevent serious consequences.

The Professional Role Fulfilment dimension covers the extent to which employees can fully perform their professional roles, whether they have the opportunity to use their knowledge and skills, whether they have to perform unnecessary additional tasks, and whether the boundaries of their roles and responsibilities are clear. In situations where there is a shortage of staff, employees often have to perform a wider range of tasks. For example, nurses may be forced to perform the tasks of sanitary workers, administrative staff, or doctors’ assistants, which goes beyond the scope of their direct role [59,83]. Similarly, a heavy workload can mean nurses are unable to devote the optimal amount of time to each patient, leading to internal dissatisfaction with the quality of their work [84]. International studies on role conflict and ambiguity demonstrate that these factors directly impact job satisfaction. When staff have unclear roles or conflicting responsibilities, job satisfaction decreases and stress increases [85]. In the context of Latvian hospitals, nurses often have to care for a large number of patients while also completing documentation, resolving logistical issues, etc., which negatively impacts their direct care duties. Such situations create a role conflict: formally, nurses are expected to provide empathetic, high-quality care, but in reality, they are forced to work in a ‘conveyor belt’ manner [86]. Data collected by SRAQ-HP on the extent to which staff feel restricted in performing their professional duties provides important information for management. For example, if the majority of nurses in a department indicate that they are unable to perform their work to a high standard due to a lack of time, this suggests a systemic issue that requires attention, whether through increasing staff numbers, improving process organisation, or reviewing the scope of nurses’ roles. In short, the Professional Role Fulfilment dimension helps us understand how healthy the work environment is from a professional point of view: do employees feel proud of their work, or are they frustrated and demotivated because they are unable to work to the best of their ability?

The third important dimension is Organisational and Managerial Support. This dimension covers staff perceptions of the extent to which management and immediate superiors support them in their daily work, in crisis situations, and in their professional development. Good management support is evident in factors such as reasonable workload distribution, addressing employee concerns, providing regular feedback, recognising professional achievements, and adopting an empathetic approach [87,88]. Several international studies confirm [87,88] that management style and support are critical factors influencing staff retention and satisfaction.

Unfortunately, Latvian hospitals are often criticised for a lack of communication and support at management level. For example, staff opinion is not taken into account when schedules are drawn up, employees do not feel safe reporting problems, and good work results go unnoticed [89]. SRAQ-HP survey data on the Managerial Support dimension enables this previously difficult-to-measure aspect to be assessed quantitatively. If the survey shows a low rating for management support, this should be a serious red flag for the institution’s management as it may indicate poor internal communication, inconsistent management practices between departments, or even an increased risk of bullying. Overall, when integrated with the other two dimensions, the Managerial Support dimension provides a more complete picture. Even at high workload, good management support can mitigate the negative effects [85]. Conversely, poor management can exacerbate the situation, even at moderate workload. Therefore, the strength of SRAQ-HP lies in its integrated assessment of these three dimensions (workload, role, and support). Together, they reveal where workload problems are most acute, whether these are exacerbated by role or organisational problems, and whether management is able to compensate for difficulties.

In summary, the discussion on the integration of the SRAQ-HP dimensions shows that the tool addresses both the ‘hard’ and ‘soft’ aspects of the workforce: workload and psychosocial factors, respectively. This approach aligns with the contemporary understanding of the quality of the working environment in healthcare, where staff well-being is influenced by a complex set of factors beyond just salaries or position numbers [90]. Together, data on subjective workload, professional fulfilment, and management support tell the story of how our nurses, doctors, and medical assistants are doing on a daily basis. This story is important to ensure that the healthcare system does not rely on the illusion that ‘everything is fine’ as long as there are enough staff on paper. In reality, people’s feelings and motivation are directly linked to patient care outcomes [91]. Therefore, using the SRAQ-HP integrated approach will help develop more people-centred and sustainable human resources policies.

In summary, introducing the SRAQ-HP tool in Latvian healthcare is an important step towards systematically identifying and addressing long-standing human resource adequacy issues. The multidimensional data provided by the tool enables comparison of the situation in Latvia with international trends, as well as providing a deeper understanding of weaknesses within the system. These weaknesses range from excessive workloads and unfulfilled professional ambitions to communication gaps between staff and management. However, a more in-depth analysis of the data is required to draw detailed conclusions about the structure of the SRAQ-HP questionnaire and its measurement properties. In the future, a detailed factor analysis, i.e., statistical research will be carried out to investigate how the survey questions are grouped into dimensions and what latent factors form the constructs of staff workload and satisfaction. This analysis will provide a scientific basis for further refining the tool and interpreting the results more accurately. For instance, it will allow to ascertain whether the SRAQ-HP confirms the three intended dimensions or if additional factors emerge in practice. At this stage, we can conclude that the development of the tool and the initial results have made a significant contribution to discussions on staff policy.

In conclusion, it should be emphasised that the SRAQ-HP is not an end goal in itself, but rather a tool for promoting change. It will only realise its true potential if the data obtained is used in practice to improve the experience of both staff and patients. By working together in an evidence-based manner, we can hope to achieve long-term positive change that will benefit healthcare professionals, patients, and society as a whole [65].

## 8. Limitations

This study presents the initial development and content validation of the SRAQ-HP and should be interpreted with caution. First, although participants were drawn from different backgrounds and regions, the samples used across piloting and qualitative work were not fully representative of the Latvian healthcare workforce: several professions (e.g., mental-health nursing, outpatient care) were under-represented, and participants were predominantly female from inpatient settings in the Rīga region. This constrains external validity. To address this, the next phase will implement stratified, multicentre recruitment (by region, care setting, and role), explicitly including male practitioners and under-represented fields to enable subgroup analyses.

Second, all data were self-reported, which may favour socially desirable responding or reflect individual perceptions rather than objective conditions (e.g., professional loyalty or fear of disclosure). Third, data collection occurred in the post-pandemic period, a time of structural and emotional change in Latvian healthcare; indicators—particularly around workload and managerial support—may therefore reflect emergency-period experiences rather than stable circumstances.

Fourth, because SRAQ-HP is a new instrument grounded in multiple theoretical sources, local benchmarks are not yet available; consequently, test–retest reliability and sensitivity to change have not yet been evaluated. Fifth, factor-analytic evidence is summarised only briefly in this article; full EFA/CFA methods and results (loadings, fit indices, AVE/CR) are reported in a companion paper. Sixth, while focus groups (*n* = 10) and cognitive interviews (*n* = 6) provided rich content insights, qualitative findings cannot be generalised to the entire workforce and remain susceptible to researcher interpretation.

Seventh, criterion and construct validity have not yet been fully established. Future work will test convergent validity (e.g., with the Copenhagen Burnout Inventory, the Practice Environment Scale of the Nursing Work Index, and the Moral Distress Scale-Revised), discriminant validity (e.g., contrasts between specialties with different workload profiles such as intensive care vs. outpatient care), and criterion validity against objective organisational indicators (patient-satisfaction results, staff-turnover/sick-leave statistics, and staffing levels).

Eighth, in this initial 26-item version Working Conditions & Support is operationalised primarily as organisational/psychosocial conditions (managerial support, scheduling, peer collaboration, access to development and resources). Physical-environment aspects (space constraints, noise, ventilation, equipment availability/layout) were deliberately excluded and will be addressed in an extended module for validation in a multicentre sample, alongside analyses of convergent validity with objective indicators and differential functioning across units.

Finally, although subscale results are reported as Mean (SD) with 95% confidence intervals, we additionally provide Median (IQR) as a sensitivity descriptor given the ordinal origin of Likert items. Future research will also focus on adapting the tool for use in other languages and cultural contexts and developing versions for additional professional groups (e.g., physicians, social workers).

## Figures and Tables

**Table 1:** Three-dimensional model.

Dimension	Focus	Theoretical Framework
Staffing Adequacy and Workload	Objective and subjective workload, staff adequacy	Role Enactment [25], ICN competencies [26]
Quality of Care	Safety, Efficiency, Patient-Centredness, and Emotional Care	WHO quality indicators [26], Caring Theory [34]
Working Conditions and Support	Organisational and psychosocial support (managerial & peer support, scheduling practices, professional development, access to internal resources)	Kanter’s Empowerment Theory [24]

**Table 2:** SRAQ-HP development steps and results obtained.

Stage	Description	Results Obtained
**1. Conceptualisation**	A literature review was conducted, and 3 theoretical frameworks were selected (Kanter, the role concept, and the ICN/WHO frameworks) [24,25,26,34]. A three-dimensional model was created.	Three theoretical foundations; a three-dimensional structure was defined.
**2. Question generation**	Based on theory, literature, and interviews, 32 Likert-scale questions were developed.	An initial version of the 32 questions was created.
**3. Expert evaluation**	Ten experts evaluated the clarity, content, and usefulness of the questions; CVI was used.	Three items were removed and nine were reworded. In Round 1 (pre-revision), I-CVI values ranged 0.531–0.719; post-revision outcomes are summarised in Results (Section 4)
**4. Focus groups and cognitive interviewing**	Focus groups (N = 10) and cognitive interviews (N = 6) analysed comprehensibility and emotional resonance.	7 questions were clarified; qualitative support for the wording was obtained.
**5. Pilot study**	A version of the questionnaire containing 29 questions was tested with 35 respondents in various medical institutions; Cronbach’s α and item-total correlations were analysed.	Cronbach’s α = 0.841; the three-factor structure was provisionally supported; low-performing items were identified.
**6. Content validity assessment**	Following the pilot study, the tool was evaluated by 5 independent experts and the FVI and repeated CVI were calculated.	Independent re-review (*n* = 5) provided repeated CVI and FVI; improvements are reported in Results (Section 4)
**7. Version III of the tool**	Following the pilot study and expert feedback, the number of questions in the tool was reduced from 29 to 26.	A final version comprising 26 questions was developed.

**Table 3:** Interpretation of the assessment of staffing adequacy and workload.

Average Indicator	Assessment Level	Interpretation
4.21–5.00	High	Employees are satisfied with the distribution of the workload and the adequacy of the available resources. This promotes a positive climate and high-quality care.
3.41–4.20	Moderately high	It is mostly positive, but a slight increase in workload is possible under certain circumstances.
2.61–3.40	Moderately low	Neutral or unconvincing attitude. There is a possibility of inadequate provision of resources.
1.81–2.60	Low	This indicates staff shortages and work overload, which jeopardise staff well-being and the quality of care.
1.00–1.80	Very low	Significant structural problems. There are chronic staff shortages and a high risk of burnout.

**Table 4:** Interpretation of the assessment of quality of care according to the average score.

Average Indicator	Assessment Level	Interpretation
4.21–5.00	High	Staff resources are considered sufficient to provide quality care. There is a reduced risk of errors and an improved safety and professional environment.
3.41–4.20	Moderately high	A mostly positive assessment, but sometimes difficulty paying attention to all patients occurs.
2.61–3.40	Moderately low	The quality of care is perceived as inconsistent or rushed due to insufficient resources.
1.81–2.60	Low	There are critical problems in care provision, an increased risk of errors and an inability to comply with standards.
1.00–1.80	Very low	A crisis situation in quality of care and safety. Immediate management intervention is required.

**Table 5:** Interpretation of the assessment of working conditions and support according to the average score.

Average Indicator	Assessment Level	Interpretation
4.21–5.00	High	Effective management support, favourable environment, and sufficient resources. Employees feel valued and are able to fulfil their duties.
3.41–4.20	Moderately high	The support is mostly good, but there is room for improvement in terms of flexibility and resource provision.
2.61–3.40	Moderately low	This indicates an inadequate support system and communication issues with management.
1.81–2.60	Low	A significant lack of management support may lead to staff dissatisfaction and emotional exhaustion.
1.00–1.80	Very low	Management support is not provided. There is a high risk of burnout and staff turnover.

**Table 6:** Interpretation of overall results based on analysis of average section results.

Combination of the Section Results	Interpretation
High average score in all sections	Indicates favourable working conditions, adequate staffing levels, and high quality of care. Adequate staffing effectively supports quality care and staff satisfaction.
Low average score in one or two sections	Indicates specific areas where improvements are needed. For example, insufficient staffing or weak management support could impact the quality of care and staff well-being.
Low average score in all sections	Reveals a general problem with resources and management involvement. A comprehensive review is needed: resource planning, workload management, and support mechanisms need to be improved.

**Table 7:** Advantages and disadvantages of SRAQ-HP.

Advantages	Disadvantages
The Likert scale provides a simple and effective method of collecting data that is easy to interpret.	Responses may be influenced by personal feelings and current working conditions, which can vary from shift to shift.
This survey has been designed to be used regularly to monitor changes in staff satisfaction and resource availability over time.	The questionnaire may not cover all personal perspectives and individual circumstances.
The questionnaire covers key factors that influence staff satisfaction, workload and quality of care.	A thorough analysis of the average scores is necessary to clearly identify dimensions

**Table 8:** Expert CVI results.

Item No.	Ex.1.	Ex.2.	Ex.3.	Ex.4.	Ex.5.	Ex.6.	Ex.7.	Ex.8.	Ex.9.	Ex.10.	SUM	CVI
**1.**	1	1	1	1	1	1	1	1	1	1	10	1
**2.**	1	0	1	1	1	0	1	1	1	1	8	0.8
**3.**	0	0	0	0	1	1	1	0	0	0	3	0.3
**4.**	0	0	1	1	1	0	0	1	0	1	5	0.5
**5.**	0	1	1	0	0	0	1	1	1	0	5	0.5
**6.**	1	1	1	0	1	1	1	1	1	1	9	0.9
**7.**	1	0	0	1	1	0	1	0	1	1	6	0.6
**8.**	1	1	1	1	1	1	1	1	1	1	10	1
**9.**	1	1	1	1	1	1	0	1	0	1	8	0.8
**10.**	0	0	1	1	1	0	1	0	1	1	6	0.6
**11.**	1	1	1	1	1	1	1	1	1	1	1	1
**12.**	1	1	1	0	0	0	0	1	0	0	4	0.4
**13.**	0	1	1	1	1	1	1	1	1	1	9	0.9
**14.**	1	1	1	1	1	1	1	1	1	1	10	1
**15.**	1	1	1	0	1	1	1	1	1	1	9	0.9
**16.**	0	1	1	1	1	1	0	0	1	0	6	0.6
**17.**	0	0	1	1	1	0	1	1	1	1	7	0.7
**18.**	1	0	1	0	0	0	1	1	0	0	4	0.4
**19.**	0	0	0	0	0	0	0	0	0	0	0	0
**20.**	0	0	1	1	1	1	1	1	0	1	7	0.7
**21.**	1	1	1	1	1	1	1	1	1	1	10	1
**22.**	1	1	1	1	1	0	1	0	1	1	8	0.8
**23.**	0	1	0	0	1	1	1	1	1	1	7	0.7
**24.**	1	1	1	0	1	1	1	1	1	1	9	0.9
**25.**	0	1	1	1	1	1	1	1	1	1	9	0.9
**26.**	0	1	1	0	0	1	1	1	0	1	6	0.6
**27.**	1	1	0	1	1	0	1	1	1	1	8	0.8
**28.**	1	1	1	1	0	0	1	1	1	1	8	0.8
**29**	1	1	0	1	1	1	0	1	1	1	8	0.8
**30.**	0	0	0	0	0	0	0	0	0	0	0	0
**31.**	0	0	0	0	0	0	0	0	0	0	0	0
**32.**	0	0	0	0	0	0	0	0	0	0	0	0
**CVI**	0.531	0.625	0.719	0.594	0.719	0.531	0.719	0.719	0.656	0.719		0.653

**Table 9:** Reaction and discrimination indices (*n* = 35).

	Mean (Reaction Index)	SD	Sum	Corrected Item-Total Correlation	Scale Mean if Item Deleted	Squared Multiple Correlation	Cronbach’s Alpha	Cronbach’s Alpha Based on Dimensions
**1**	2.26	1.120	79	0.270	88.74	0.931	0.839	0.620
**2**	2.6	1.090	91	0.346	88.40	0.920	0.837	
**3**	2.09	1.095	73	0.181	88.91	0.938	0.841	
**4**	2.89	1.323	101	0.319	88.11	0.858	0.838	
**5**	2.2	1.208	77	0.139	88.80	0.930	0.843	
**6**	4.11	1.051	144	0.460	86.89	0.916	0.833	
**7**	3.03	1.294	106	0.258	87.97	0.842	0.840	
**8**	2.71	1.178	95	0.403	88.29	0.839	0.835	
**9**	3.80	1.431	133	0.444	87.20	0.939	0.833	
**10**	3.54	1.291	124	0.256	87.46	0.914	0.840	
**11**	3.57	1.290	125	0.580	87.43	0.915	0.828	0.817
**12**	3.26	1.291	114	0.062	87.74	0.910	0.846	
**13**	4.03	1.014	141	0.631	86.97	0.777	0.829	
**14**	4.14	1.061	145	0.510	86.86	0.826	0.832	
**15**	4.09	1.173	143	0.318	86.91	0.958	0.837	
**16**	3.89	1.132	136	0.116	87.11	0.946	0.844	
**17**	4.03	1.071	141	0.339	86.97	0.943	0.837	
**18**	4.09	1.095	143	0.104	86.91	0.838	0.844	
**19**	3.57	1.170	125	0.274	87.13	0.955	0.839	
**20**	2.51	1.095	88	0.458	88.49	0.908	0.833	0.888
**21**	2.14	1.167	75	0.544	88.86	0.849	0.830	
**22**	2.77	1.239	97	0.558	88.23	0.901	0.829	
**23**	3.26	1.314	114	0.523	87.74	0.821	0.830	
**24**	2.94	1.083	103	0.399	88.06	0.909	0.835	
**25**	3.51	1.121	123	0.491	87.49	0.891	0.832	
**26**	2.51	1.197	88	0.489	88.49	0.978	0.832	
**27**	2.23	0.973	78	0.386	88.77	0.789	0.836	
**28**	3.0	1.350	105	0.545	88.00	0.924	0.829	
**29**	2.23	1.140	78	0.108	88.77	0.800	0.844	

**Table 10:** SRAQ-HP scale.

Nr.	Item
*Staffing Adequacy and Workload*	
1.	There are enough staff in our department during shifts to provide the necessary patient care.
2.	The workload is reasonable and allows me to perform my care tasks to the fullest extent.
3.	Our department has sufficient staff to provide the necessary care for patients.
4.	I don’t have to take on the duties of other colleagues due to staff shortages.
5.	I am able to devote sufficient time to the individual needs of each patient.
6.	There are enough staff members to ensure that all tasks are completed fully and on time.
7.	My department has clearly defined staff priorities to help manage the workload, especially during overload periods.
8.	We have enough staff to avoid work overload.
9.	Our department always has enough staff to complete daily tasks.
*Quality of Care*	
10.	The adequacy of staffing in our department significantly improves the quality of care and patient safety.
11.	The current number of staff ensures that all care tasks are performed adequately.
12.	Patients in our department receive high-quality care, provided there are sufficient staff resources.
13.	I can provide safe care when there are enough staff in the department.
14.	Staff shortages negatively impact patient satisfaction and care outcomes.
15.	I have enough time to provide emotional support to patients.
16.	Work overload does not affect my ability to provide quality care.
*Working Conditions and Support*	
17.	The hospital provides sufficient support and resources to perform care tasks effectively.
18.	Management responds actively to issues of work overload and provides support when needed.
19.	The hospital offers opportunities to acquire additional skills and knowledge to help manage the workload.
20.	I often receive the necessary support from colleagues, which improves the quality of my work.
21.	The work schedule in our department is designed to avoid staff shortages during shifts.
22.	I feel that the schedule is flexible and takes my personal needs into account.
23.	Management takes measures to ensure the health and safety of employees at work.
24.	Management is often involved in responding to suggestions from employees on how to improve work resources.
25.	Our department has support mechanisms in place, such as the opportunity to consult with colleagues or management, in case psychological support is needed due to staff shortages.
26.	There are incentive programmes and bonuses to help improve staff retention and satisfaction.

**Table 11:** CVI results from *n* = 5 experts.

Item No.	Ex.1.	Ex.2.	Ex.3.	Ex.4.	Ex.5.	SUM	CVI
**1.**	1	1	1	1	1	5	1
**2.**	1	0	1	1	1	4	0.8
**3.**	1	1	1	1	1	5	1
**4.**	1	1	1	1	1	5	1
**5.**	1	1	1	1	1	5	1
**6.**	1	1	1	1	1	5	1
**7.**	1	1	0	1	1	4	0.8
**8.**	1	1	1	1	1	5	1
**9.**	1	1	1	1	1	5	1
**10.**	1	1	1	1	1	5	1
**11.**	1	1	1	1	1	5	1
**12.**	1	1	1	1	1	5	1
**13.**	0	1	1	1	1	4	0.8
**14.**	1	1	1	1	1	5	1
**15.**	1	1	1	0	1	4	0.8
**16.**	1	1	1	1	1	5	1
**17.**	1	1	1	0	1	4	0.8
**18.**	1	1	1	1	1	5	1
**19.**	1	0	1	1	1	4	0.8
**20.**	1	1	0	1	1	4	0.8
**21.**	1	1	1	1	1	5	1
**22.**	1	1	1	1	1	5	1
**23.**	1	1	1	1	0	4	0.8
**24.**	1	1	1	0	1	4	0.8
**25.**	1	1	1	1	1	5	1
**26.**	1	1	1	1	1	5	1
**CVI**	0.962	0.923	0.923	0.884	0.962		0.931

**Table 12:** FVI from *n* = 5 experts.

Item	Ex.1.	Ex.2.	Ex.3.	Ex.4.	Ex.5.	SUM	FVI
**1.**	1	1	1	1	1	5	1
**2.**	1	1	1	1	1	5	1
**3.**	1	1	1	1	1	5	1
**4.**	1	1	1	1	1	5	1
**5.**	1	1	1	1	1	5	1
**6.**	1	1	1	1	1	5	1
**7.**	1	1	1	1	1	5	1
**8.**	1	1	1	1	1	5	1
**9.**	1	1	1	1	1	5	1
**10.**	1	1	1	1	1	5	1
**11.**	1	1	1	1	1	5	1
**12.**	1	0	1	1	1	4	0.8
**13.**	1	1	1	1	1	5	1
**14.**	1	1	1	0	1	4	0.8
**15.**	1	1	1	1	1	5	1
**16.**	1	1	1	1	1	5	1
**17.**	1	1	1	1	1	5	1
**18.**	1	1	1	1	1	5	1
**19.**	1	1	1	1	1	5	1
**20.**	1	1	1	1	1	5	1
**21.**	1	1	1	1	1	5	1
**22.**	1	1	1	1	1	5	1
**23.**	1	1	1	1	1	5	1
**24.**	1	1	0	1	1	4	0.8
**25.**	1	1	1	1	1	5	1
**26.**	1	1	1	1	1	5	1
**FVI**	1	0.961	0.961	0.961	1		0.976

## Data Availability

The datasets produced and examined in this study can be obtained from the corresponding author upon a reasonable request. All data generated or analyzed during this study are provided within the published article. The data utilized in this study is confidential.

## Abbreviations

The following abbreviations are used in this manuscript:

SRAQ-HP	Staff Resource Adequacy Questionnaire for Healthcare Professionals
CVI	Content Validity Index
FVI	Face Validity Index
NPM	Nurse-to-Patient Mix
COVID-19	Coronavirus Diseas 2019
ASCOP	Association of Cancer Physicians
NWI	Nursing Working Index
NPC	Nurse-to-Patient ratio
ICN	International Council of Nurses
WHO	World Health Organization
n	Number
GDPR	General Data Protection Regulation
RSU	Riga Stradiņš University
CBI	Copenhagen Burnout Inventory
PES-NWI	Practice Environment Scale of the Nursing Work Index
MBI	Maslach Burnout Inventory
ROC	Receiver Operating Characteristic
FTE	Full-Time Equivalent
Min	Minimum
Max	Maximum
SD	Standard Deviation
SMC	SMC
S-CVI	Summa of CVI
EU	European Union
RAFAELA	(Finnish system name; no acronym expansion—officially a proper name)
PAONCIL	Professional Assessment of Optimal Nursing Care Intensity Level
OECD	Organisation for Economic Co-operation and Development
THL	Finnish Institute for Health and Welfare (Terveyden ja hyvinvoinnin laitos)
AMOS	Analysis of Moment Structures (IBM SPSS Amos software, Version 28.0 (IBM Corp., Armonk, NY, USA))
SPSS	Statistical Package for the Social Sciences
